# Unraveling Thermal Interactions in Lanthanide-Doped Phosphors: A Frequency-Domain Analysis Approach

**DOI:** 10.1021/acs.jpclett.5c04010

**Published:** 2026-03-04

**Authors:** Manuel Romero, Victor Castaing, Daniel Rytz, Gabriel Lozano, Hernán Míguez

**Affiliations:** † Institute of Materials Science of Seville, Spanish National Research Council − University of Seville, C. Américo Vespucio 49, 41092 Seville, Spain; ‡ BREVALOR Sàrl, 1669 Les Sciernes, Switzerland

## Abstract

Ensuring the thermal reliability of luminescent materials is a key requirement for next-generation lighting, display, and sensing technologies. The intricate interplay of thermal crossover and thermal ionization in lanthanide-doped phosphors often obscures their individual contributions. We present a frequency-domain photoluminescence analysis that disentangles these competing mechanisms. Using single crystals of SrAl_2_O_4_:Eu^2+^,Dy^3+^ (SAO:Eu,Dy) and (Gd_0.33_Y_0.67_)_3_Al_2.4_Ga_2.6_O_12_:Ce^3+^,Cr^3+^ (GYAGG:Ce,Cr) as model systems, we extract temperature-dependent trapping efficiencies and decay rates by analyzing the phase and amplitude response of luminescence under modulated excitation. Our approach reveals distinct signatures of thermal ionization and enables the direct quantification of ionization barriers and crossover rates. We demonstrate that SAO:Eu,Dy exhibits dominant trapping behavior with high ionization efficiency, while GYAGG:Ce,Cr shows significant competition between ionization and crossover. This method provides a powerful framework for resolving overlapping quenching pathways and offers new insights for the design of thermally robust luminescent materials.

## Graphical abstract

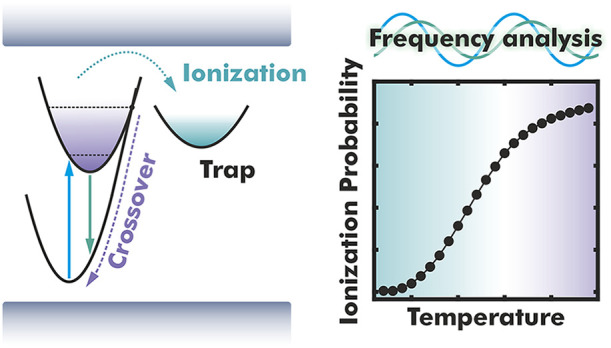

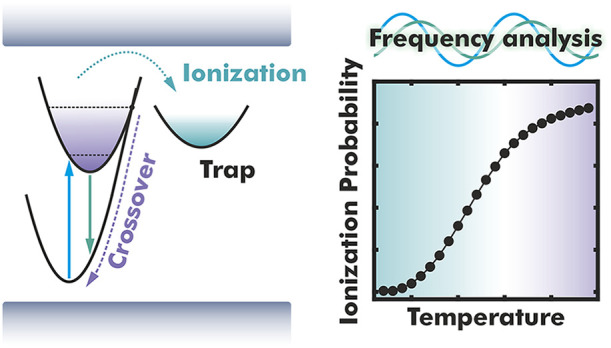

Understanding thermal quenching in luminescent materials is crucial for advancing our knowledge of energy transfer and charge carrier dynamics in solids.^1–5^ In lanthanide-doped phosphors, thermal quenching arises primarily from two competing nonradiative processes: thermal crossover and thermal ionization.^6–9^ Thermal crossover involves multiphonon-assisted promotion of charges from an excited state to higher vibrational levels within the emitting cation, where the charges decay nonradiatively.^8,9^ This process becomes increasingly probable as the thermal population of vibrational levels (VL) rises. Thermal ionization involves promoting excited electrons into the conduction band (CB) of the host. Both processes lead to luminescence quenching as the charge density of the excited state decreases. However, charges in the CB resulting from thermal ionization may become trapped at defect sites. Such trapped charge carriers can eventually return to the excited state, resulting in delayed recombination and long-lived afterglow, also known as persistent luminescence (PersL).^8–11^

Thermal quenching in complex garnets (Y_3_Al_5‑x_Ga_*x*_O_12_:Pr^3+^) have been associated with thermally activated crossover or thermal ionization depending on the Ga content.^9^ In addition, the temperature-dependent emission behavior of highly stable Y_3_Al_5_O_12_:Ce^3+^ (YAG:Ce) is primarily determined by thermal ionization with an onset temperature of luminescence quenching close to 600 K.^10^ Similarly, thermally assisted electron transfer from Eu^2+^ to Dy^3+^ in SrAl_2_O_4_:Eu^2+^,Dy^3+^ (SAO:Eu,Dy) has been identified as another manifestation of thermal ionization and the overlaying mechanism in PersL materials.^12,13^ These examples illustrate that the response of a luminescent material is modulated by both thermal crossover and thermal ionization processes, which often coexist and interact over broad temperature ranges. Despite their importance in limiting efficiency and thermal stability, distinguishing and quantifying the individual contributions of these processes remains a significant challenge for the design of thermally stable luminescent materials. Conventional analysis relies on characterizing photoluminescence (PL) intensity as a function of temperature. While this yields quenching curves that can be used to extract activation energies, the analysis fails to provide mechanistic insight. This is because analyzing these curves cannot distinguish the overlapping effects of crossover and ionization, nor can it reveal the intrinsic material parameters that govern carrier dynamics. Although more advanced characterization tools have proven effective in investigating thermal interactions,^8–11,14^ precisely assessing the relative contributions of coexisting thermal quenching processes remains challenging.

Modulation-based techniques were initially proposed for determining the lifetime of light sources and quickly became relevant for analyzing fluorescent lifetime imaging microscopy.^15,16^ Recently, these techniques have been employed for studying charge trapping processes in PersL materials and metal halide perovskites.^17–19^ In this work, we use frequency-domain analysis to investigate the thermal properties of two single-crystal phosphors, SAO:Eu,Dy and (Gd_0_._33_Y_0_._67_)_3_Al_2_._4_Ga_2_._6_O_12_:Ce^3+^,Cr^3+^ (GYAGG:Ce,Cr), two examples of PersL materials in which thermal ionization and crossover occur. By probing the luminescent signal under modulated excitation, we extract quantitative information on the trap depth or the ionization barrier, and the relative contribution of thermal ionization to the overall excited state decay dynamics. This approach enables a direct comparison of the competing quenching pathways and provides a framework for understanding and engineering thermal stability in luminescent materials.

Luminescence in lanthanide ions may involve transitions between 5d and 4f states. Thermal crossover takes place when the temperature is high enough to promote electrons from the 5d states to a higher VL from which they decay nonradiatively to the 4f ground state, as depicted in Figure 1a. Depending on the particular combination of emitter and host, the 5d excited state and the 4f ground state may lay within the bandgap, as it occurs for Ce^3+^ in YAG:Ce or Eu^2+^ in SAO:Eu,Dy,^10,12^ while in others, 5d levels are closer to the CB of the host, as for Ce^3+^ in Y_3_Ga_5_O_12_:Ce^3+^ (YGG:Ce).^8,9^ Thus, thermal energy can also favor the ionization of emitting cations and the subsequent migration of electrons through the CB, which increases the probability of charge trapping in structural defects of the lattice (see Figure 1a). Both thermal ionization and thermal crossover favor the nonradiative emptying of the 5d excited state. This results in reduced luminescence intensity and quantum yield. The standard analysis of these phenomena based only on the dependence of PL intensity on temperature over time (i.e., the quenching curve), cannot distinguish the contributions of these mechanisms unless additional measurements are performed.^8–11,14^ Nevertheless, when charge trapping follows thermal ionization, the rate at which the excited state empties increases, and charge carriers accumulate in traps that can eventually release and recombine in the luminescent center. This gives rise to the delayed luminescence signal typically associated with PersL and enables the separation of the two thermal quenching mechanisms.

**Figure 1:**
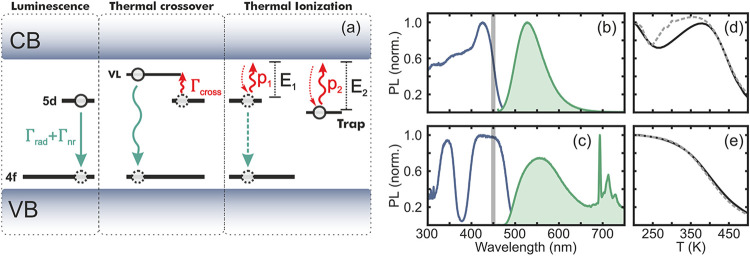
(a) Schematic of the luminescence, thermal crossover and thermal ionization processes. Red solid arrows correspond to thermally activated processes i.e., thermal crossover to higher vibrational levels, electron migration to the CB or charge detrapping. (b,c) Excitation (blue) and emission (green) spectra for SAO:Eu,Dy (b) and GYAGG:Ce,Cr (c). (d,e) PL intensity as a function of temperature and excitation density (dashed lines correspond to 0.04 mW/cm^2^ and solid lines correspond to 4 mW/cm^2^) for SAO:Eu,Dy (b) and GYAGG:Ce,Cr (c).

To illuminate this complex interaction, we chose two well-known PersL phosphors in their single crystal form: The first is GYAGG:Ce,Cr, representing Ce^3+^-doped garnets characterized by efficient PL. The second is SAO:Eu,Dy, which is an example of a bright and efficient afterglow at room temperature. Excitation and emission spectra are shown in Figure 1b,c. We measure the quenching curves of these materials and observe a 50% drop in intensity at *T*_50%_ ∼ 400 K for GYAGG:Ce,Cr, and at *T*_50%_ ∼ 450 K for SAO:Eu,Dy (Figure 1d,e). Both materials show an excitation intensity dependence of the normalized quenching curves which cannot be explained attending to the thermal crossover mechanism alone. Additionally, SAO:Eu,Dy exhibits a modulation in the intensity at temperatures below *T*_50%_, which we attribute to the interplay between the dynamics of the excited and trap states associated with PersL in SAO:Eu,Dy.

Recently, a frequency domain analysis has been applied to characterize the delayed luminescence in PersL materials, which allows to quantify the intrinsic rates of PersL phosphors.^19^ In particular, the trapping efficiency (β) is defined as

1 $$\beta \left(T\right)=\frac{p_{1}\left(T\right)}{\Gamma_{rad}+\Gamma_{nr}+\Gamma_{cross}\left(T\right)+p_{1}\left(T\right)}$$

Note that this magnitude is temperature dependent. The trapping rate, *p*_1_, corresponds to the trap-assisted thermal ionization rate, Γ_*cross*_ is the thermal crossover rate, Γ_*rad*_ is the radiative decay rate, and Γ_*nr*_ is associated with any additional nonradiative rate such as energy transfer between emitting cations. eq 1 explicitly shows that thermal ionization and thermal crossover are competing mechanisms. To disentangle the contribution of each process to the luminescence, we studied the temperature dependence of the frequency response of SAO:Eu,Dy and GYAGG:Ce,Cr single crystals under low intensity blue light (450 nm) excitation, which is shown in Figure 2. Briefly, when measuring the frequency response of a photoluminescent material it is possible to directly access the intrinsic characteristic rates of the system.^19^ The transfer function H is given by

2 $$H\left(\nu \right)=H_{r}\left(\nu \right)-iH_{i}\left(\nu \right)=\frac{A_{emi}^{*}}{A_{exc}^{*}}\left(\cos\Delta \phi -i\;\sin\Delta \phi \right)$$

where *A*_emi_^*^ and *A*_exc_^*^ correspond, respectively, to the modulation amplitudes of the luminescence and the excitation signals normalized to their average values, and Δϕ is the phase difference between the luminescence and excitation signals.

**Figure 2:**
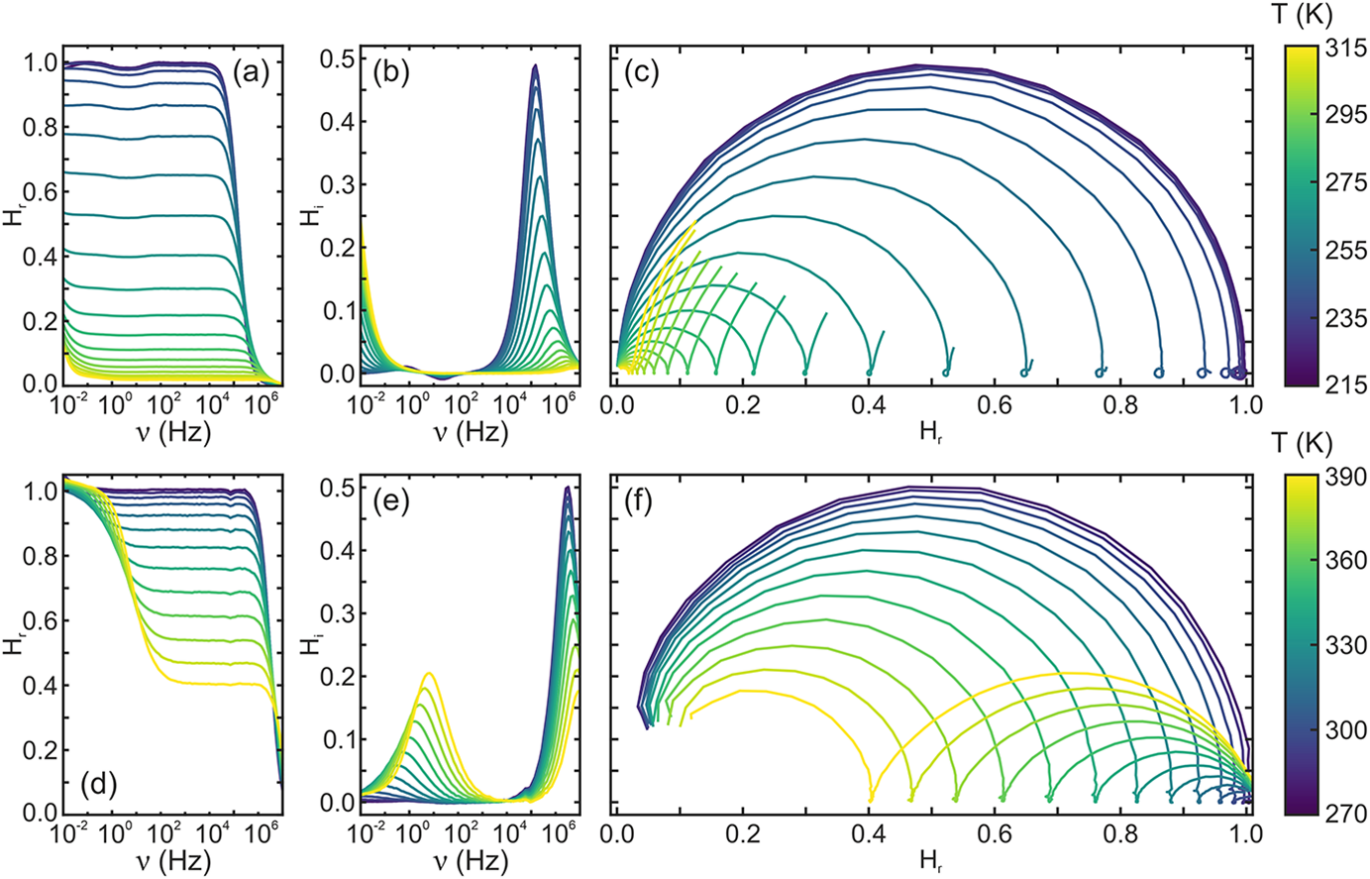
Temperature dependent frequency domain analysis of a SAO:Eu,Dy (a–c) and a GYAGG:Ce,Cr (d–f) single crystal. Real (a,d) and imaginary (b,e) part of the transfer function as a function of excitation modulation frequency. (c,f) Nyquist plot of the transfer function at different temperatures.

In the absence of thermal interactions (*p*_1_ = 0; Γ_*cross*_ = 0), the dynamics of the luminescent material are governed by the total decay rate (Γ_*tot*_), given by the sum of Γ_*rad*_, and Γ_*nr*_. Specifically, a narrow peak should appear in the imaginary part of the transfer function at a frequency equal to (Γ_*rad*_ + Γ_*nr*_)/2π, and a single semicircle of 0.5 radius would be present in the Nyquist plot (*H*_*r*_ vs *H*_*i*_). At low temperatures (below 240 K for SAO:Eu,Dy, and below 290 K for GYAGG:Ce,Cr) the materials under study are unaffected by thermal crossover or ionization, making their frequency response resemble that of a single decay rate luminescent material. For SAO:Eu,Dy, the peak appears at 0.14 MHz, corresponding to a decay lifetime of 1.1 μs. In GYAGG:Ce,Cr, the peak appears at 3.1 MHz, corresponding to a lifetime of 51 ns. For both materials, the reduced peak width indicates that a single decay rate is present rather than a distribution of rates, as expected for single crystals. Conventional time-dependent PL measurements confirm a single-exponential behavior, with lifetimes that are in excellent agreement with values extracted from the frequency analysis (see Supporting Information). Interestingly, when the luminescence of a material is the result of a more complex dynamic process, its frequency response features distinct lobes in the Nyquist plot, associated with *H*_*i*_ peaks. In the case of delayed luminescence caused by thermal ionization, two peaks are expected. One appears at high frequencies and corresponds to the total emptying rate of the excited state (Γ_*rad*_ + Γ_*nr*_ + Γ_*cross*_ + *p*_1_). The other peak, which appears at low frequencies, relates to the detrapping rate. It is far apart in the frequency response because p_2_ is orders of magnitude smaller than Γ_*tot*_. The height of the high- and low-frequency peaks, the corresponding H_r_ value, and the length of each lobe in the Nyquist plot provide direct insight into the contribution of fast and slow processes to luminescence and trapping efficiency. When these processes become comparable, however, the frequency response may exhibit overlapping features, which complicates the extraction of the system’s characteristic rates. This may be relevant for materials dominated by shallow traps or those with broad trap distributions.

As the temperature increases, so does the low-frequency peak associated with delayed luminescence for both materials under study. Concurrently, the height of the high-frequency peaks decreases and shifts toward higher frequencies (see Figure 2). Consequently, the low-frequency lobe enlarges, suggesting an increase in β with T, as more electrons are captured by traps instead of decaying faster from the 5d level. However, despite their similar general behavior, clear differences are observed. First, the low-frequency peak in SAO:Eu,Dy appears at lower frequencies, indicating a lower detrapping rate. Second, the size of the low-frequency lobe in GYAGG:Ce,Cr increases more slowly with temperature, suggesting a different thermal barrier for trapping as we will discuss next.

To further investigate this, Figure 3a shows the calculated β extracted from the Nyquist plots displayed in Figures 2c and 2f. The results are shown as open symbols for SAO:Eu,Dy and as filled symbols for GYAGG:Ce,Cr. Our analysis confirms that both materials exhibit an increase in β with temperature, as expected when thermal ionization becomes dominant. The value of β reaches 50% at 260 K for SAO:Eu,Dy and at 380 K for GYAGG:Ce,Cr. This is close to *T*_50%_, which suggests that trapping and thermal crossover coexist within the same temperature range. As depicted in Figure 1a, PersL materials can be modeled as a three-level system consisting of ground, excited, and trap energy levels. If we consider Γ_*cross*_ as a nonradiative rate and disregard charge transport through the CB, the high frequency H_i_ peak (2π*ν*_*H*_) can be associated with the emptying rate of the excited state (Γ_*rad*_ + Γ_*nr*_ + Γ_*cross*_ + *p*_1_). This enables us to use eq 1 to extract p_1_ and Γ_*tot*_ (defined as Γ_*rad*_ + Γ_*nr*_ + Γ_*cross*_) from the measured β value at each temperature:

3 $$p_{1}\left(T\right)=2\pi \nu_{H}\beta$$

4 $$\Gamma_{tot}\left(T\right)=2\pi \nu_{H}-p_{1}=2\pi \nu_{H}\left(1-\beta \right)$$

**Figure 3:**
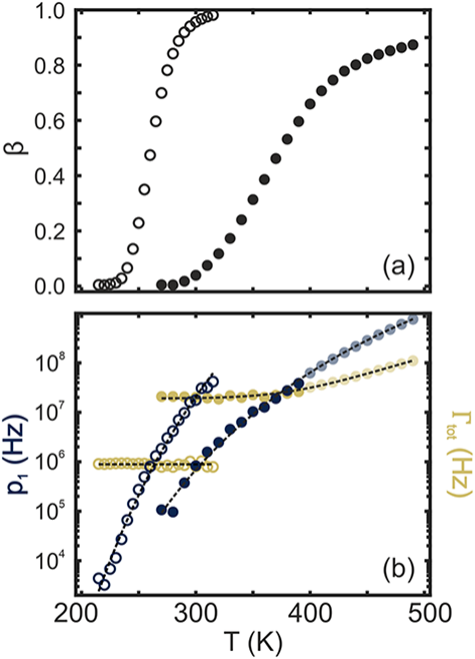
Trapping efficiency (a), trapping rate and total decay rate (b) of a SAO:Eu,Dy (open symbols) and a GYAGG:Ce,Cr (filled symbols) single crystal. Lighter points correspond to extrapolated values using eq 1, the values measured of β and the fitting parameters of p1.

This allows disentangling the contributions of thermal crossover and thermal ionization. The results are shown in Figure 3b as open symbols for SAO:Eu,Dy and as filled symbols for GYAGG:Ce,Cr. We observe distinct temperature dependencies of *p*_1_ and Γ_*tot*_ for each material under study. Although this analysis cannot distinguish between the individual contributions to Γ_*tot*_, it enables the measurement of the thermal evolution of Γ_*cross*_, provided that Γ_*rad*_ + Γ_*nr*_ are considered to be temperature independent. Specifically, thermal crossover is absent in SAO:Eu,Dy within the studied temperature range, as evidenced by the constant value of Γ_*tot*_ (∼1 MHz, see Figure 3b). However, GYAGG:Ce,Cr shows a clear increase in Γ_*tot*_ at temperatures higher than 380 K. Our analysis confirms that the competition between thermal crossover and thermal ionization in GYAGG:Ce,Cr prevents β from reaching values as high as those in SAO:Eu,Dy, which has nearly 100% trapping efficiency at room temperature.

Further analysis of the results can reveal more about the link between material parameters and photophysical performance. It was possible to fit the *p*_1_ values obtained experimentally to the Arrhenius equation

5 $$p_{1}\left(T\right)=s_{1}e^{-E_{1}/k_{B}T}$$

where *s*_1_ is the frequency factor for thermal ionization, E_1_ is the energy barrier for trapping, and *k*_*B*_ is the Boltzmann constant.^20,21^ Fits using eq 5 are shown as dashed lines in Figure 3b, which fairly agree with the experimental data. The values for *s*_1_ and *E*_1_ are included in Table 1. Although SAO:Eu,Dy was found to have a greater trapping barrier than GYAGG:Ce,Cr (0.59 eV vs ∼0.47 eV), it also has a higher trapping efficiency. This is because the s_1_ value estimated for SAO is significantly larger than that of GYAGG:Ce,Cr (1.8·10^17^ *Hz* vs 4.9·10^13^ *Hz*). These findings suggest that the ionization mechanism in these materials may either be different or the concentration of accessible traps may vary greatly.

**Table 1:** Frequency Factor (s_1_) and Energy Barrier for Thermal Ionization (E_1_) Extracted from Figure 3b^a^

Material	*s*_1_ (Hz)	*E*_1_ (eV)	*s*_2_ (Hz)	*E*_2_ (eV)
SAO:Eu,Dy	(1.8 ± 1.0) × 10^17^	0.59 ± 0.01	2.6 × 10^10^	0.66
GYAGG:Ce,Cr	(4.9 ± 3.1) × 10^13^	0.47 ± 0.02	3.7 × 10^11^	0.73

a Frequency factor for detrapping (s_2_) and trap depth (E_2_) extracted from the fittings of the frequency response are also included.

To the best of our knowledge, reported values for the trapping barrier (E_1_) are scarce. While a comparable ionization barrier (0.50 eV) has been reported for YAG:Ce,Cr,^10^ experimental estimates of the trapping frequency factor (s_1_) have not. This emphasizes the difficulty of obtaining these values with conventional techniques and the potential of the frequency-domain analysis for evaluating trapping parameters.

To shed more light on the trapping-detrapping mechanism, we calculate E_2_ and p_2_ in these materials by fitting the experimental frequency spectra.^19,22^ In the calculations, we employed a trap depth distribution, i.e. a set of values for E_2_, assuming that p_2_ follows the expression

6 $$p_{2}\left(T\right)=s_{2}e^{-E_{2}/k_{B}T}$$

where *s*_2_ is the frequency factor for detrapping, which may be different from *s*_1_. Fits are shown in the Supporting Information (see Figure S2), with parameters consistent with those directly extracted from the measurements (see Table S1 of the Supporting Information). Although we use a local model, which typically assumes that a trap interacts only with its nearest recombination center (as occurs when Eu^2+^ ions are directly excited in SAO:Eu,Dy), our theoretical analysis also helps us to understand the trapping mechanisms of more complex systems in which the CB is involved in the trapping process (as in GYAGG:Ce,Cr).^8,10,14^ In this case, the aforementioned p_1_ values can be interpreted as resulting from transfer from the luminescent center to the CB, transport within the band, and subsequent capture by the trap. Note that s_1_ and s_2_ are found to be notably different, which may uncover distinct trapping and detrapping mechanisms in these materials, e.g., tunneling or band assisted. Theoretical analysis of the detrapping process shows that, although both materials have a similar E_2_, GYAGG:Ce,Cr has deeper traps (∼ 0.73 eV vs ∼ 0.66 eV) and a higher *s*_2_ value (3.7 × 10^11^ Hz vs 2.6 × 10^10^ Hz). This results in faster trap emptying. Additionally, it is noteworthy that E_2_ is higher than E_1_ in both materials, which is consistent with the classical picture of traps being deeper in energy than the 5d levels from the CB, as depicted in Figure 1a. Finally, note that these values are consistent with previous reports for similar garnets, i.e. s_2_ from 10^11^ to 10^13^ Hz and E_2_ between 0.4 and 1.0 eV,^11^ and SAO:Eu,Dy, i.e. s_2_ from 10^7^ to 10^9^ Hz and E_2_ from 0.54 to 0.86 eV.^23,24^ However, the wide dispersion of values reported for the intrinsic parameters of these systems limits direct quantitative comparisons and compromises material optimization.

In summary, our analysis yields effective trapping and detrapping rates that accurately describe the photophysics involved in long-lasting emission. Our results confirm that the origin of PersL in SAO and GYAGG is somewhat local and emphasize the distinct nature of the trapping and detrapping processes, as evidenced by their significantly different frequency factors. These results align well with the community’s current efforts to develop a comprehensive understanding of the mechanisms and processes that determine PersL.^12,25,26^ Beyond providing mechanistic insight, the frequency-domain approach offers direct access to intrinsic rates and absolute trapping efficiencies across widely separated time scales, which is difficult to achieve using conventional time-domain techniques. Our results, thus, underscore the potential of frequency analysis as a tool for establishing robust connections between materials and their photophysical properties. In this context, the frequency factors for trapping and detrapping are identified as key parameters.

In conclusion, we present a PL analysis in the frequency domain as a direct, quantitative study of thermal ionization and thermal crossover in lanthanide-doped phosphors. It has been possible to directly identify features of the thermal ionization process as a function of temperature and quantify both the trapping and thermal crossover rates of SAO:Eu,Dy and GYAGG:Ce,Cr single crystals. SAO:Eu,Dy PL is dominated by trapping, as expected in the best-performing PersL phosphor, while GYAGG is mostly dominated by thermal crossover, though trapping is also present in this material. This study paves the way for the future analysis and design of new, application-oriented phosphors, such as high-power LED phosphors with reduced thermal crossover, high-performance PersL phosphors with a low energy barrier for trapping or even antithermal quenching phosphors, which enhances afterglow intensity.

## Methods

### Materials

GYAGG:Ce,Cr and SAO:Eu,Dy single crystals were grown by a Czochralski-type process developed by BREVALOR Sàrl.

### Photophysical Characterization

An experimental setup developed in-house, referred to as *Frequency Analysis of Time Rates under Operando conditions (FARO)*, was employed in this study. Optical excitation was provided by a 200 mW blue laser operating at 450 nm, modulated through an acousto-optic modulator (Aerodiode 400FSAOM-200-0.5). The excitation power density was adjusted by means of optical neutral density filters. Signal detection was carried out using an avalanche photodiode (Thorlabs APD130A2/M) for the reference and a photomultiplier tube module (Thorlabs PMT1001/M) for the emission signal. Data acquisition was performed with a lock-in amplifier (Zurich Instruments HF2LI).

Steady-state excitation and emission spectra were collected using an Edinburgh FLS1000 spectrofluorometer. Temperature-dependent luminescence measurements were conducted with an OceanInsight Flame spectrometer. Sample temperature was controlled using a Linkam THMS600 temperature-controlled stage.

### Modeling

Analysis and fitting of the frequency-domain spectra were performed using custom MATLAB routines developed in-house, based on the analytical solutions of a three-level system rate equations, as described elsewhere.^19^

## Supplementary Material





## Data Availability

The data underlying this study are openly available in the Digital CSIC repository at https://doi.org/10.20350/digitalCSIC/18118.

## Notes

Measurements of the amplitude ratio and phase difference in the frequency domain as a function of temperature, calculations of the transfer function as a function of temperature, table of SAO and GYAGG parameters, and measurements of the PL decay (PDF)
Transparent Peer Review report available (PDF)

## §

Laboratoire de Physique de la Matière Condensée (PMC), CNRS, Ecole Polytechnique-Institut Polytechnique de Paris, 91120 Palaiseau, France
