# Supplementary material for: Unraveling Thermal Interactions in Lanthanide-Doped Phosphors: A Frequency-Domain Analysis Approach
Source: J Phys Chem Lett. 2026 Mar 4;17(11):3266–71. doi: 10.1021/acs.jpclett.5c04010 (PMC13007026; doi:10.1021/acs.jpclett.5c04010)
Supplement: Supplementary file 1 [file jz5c04010_si_001.pdf]

Supporting information of:

Unraveling Thermal Interactions in Lanthanide-Doped Phosphors: A Frequency-Domain Analysis Approach

Manuel Romero,[1,] Victor Castaing,[1,^] Daniel Rytz,[2] Gabriel Lozano,[1,*] Hernán Míguez[1]*

[1] Institute of Materials Science of Seville, Spanish National Research Council – University of Seville, C. Américo Vespucio 49, 41092, Seville, Spain.

[2] BREVALOR Sàrl, 1669 Les Sciernes, Switzerland

[^] Present address: Laboratoire de Physique de la Matière Condensée (PMC), CNRS, Ecole Polytechnique-Institut Polytechnique de Paris, 91120 Palaiseau, France.

[*] Correspondence should be addressed to MR (manuel.romero@csic.es) or GL (g.lozano@csic.es).

Amplitude ratio and phase difference spectra

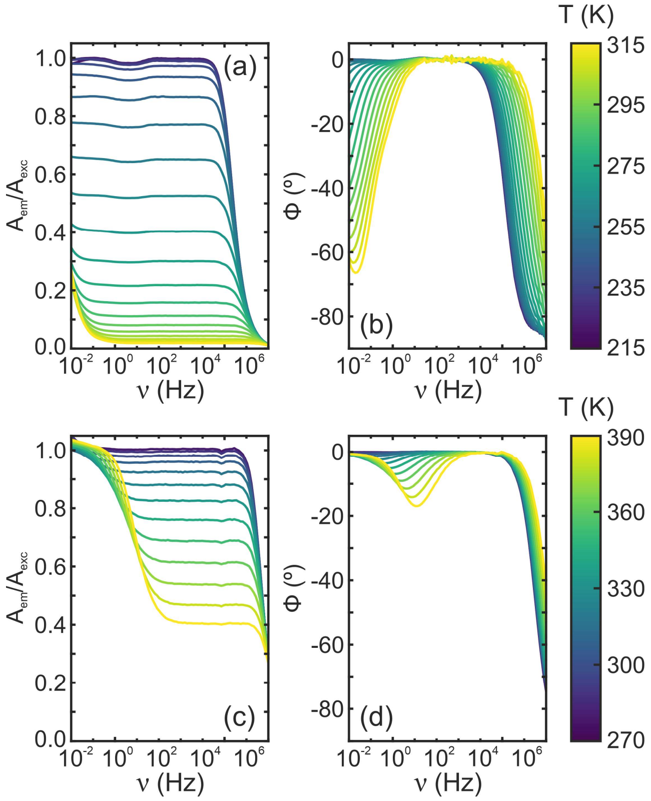

Fig. S1. Experimental amplitude ratio (a,c) and phase difference (b,d) from which the frequency response shown in Fig. 2 of the main manuscript is extracted. (a-b) Correspond to the SAO:Eu,Dy single crystal while (c-d) correspond to the GYAGG:Ce,Cr single crystal.

Calculations of the transfer function

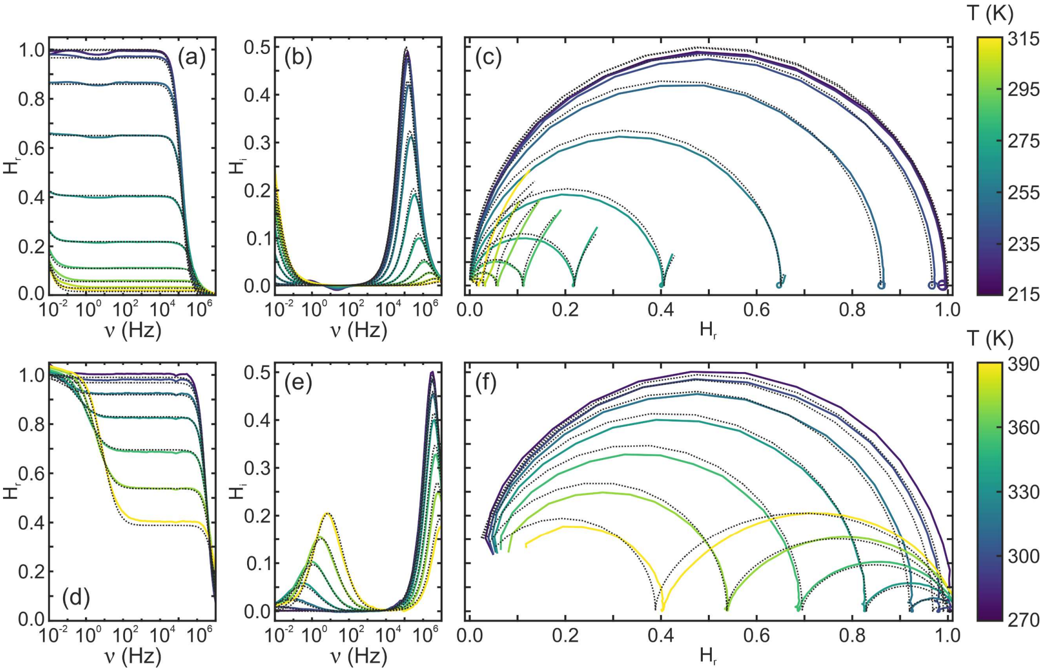

Fig. S2. Fit (dashed lines) of the experimental spectra of Fig. 2 of the main manuscript using the local model for trapping. The employed parameters are included in Table S1. (a-c) correspond to calculations for SAO:Eu,Dy while (d-f) correspond to GYAGG:Ce,Cr.

Material	p_e *(Hz)*	α	s_1 *(Hz)*	E_1 *(eV)*	s_2 *(Hz)*	E_2 *(eV)*	σ_{E_2} *(meV)*
SAO:Eu,Dy	10^{-4}	0.42	$2.95 \cdot 10^{16}$	0.54	$2.57 \cdot 10^{10}$	0.66	30
GYAGG:Ce,Cr	10^{-4}	1.00	$2.28 \cdot 10^{12}$	0.47	$3.68 \cdot 10^{11}$	0.73	42

Table S1. Frequency factor and energy barrier for thermal ionization extracted from Fig.3b,d.

Time-domain photoluminescence decay measurements

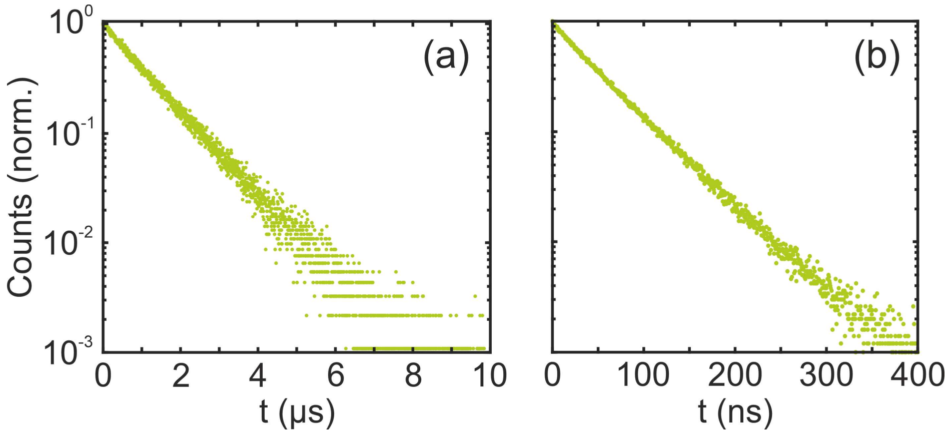

Fig. S3. Photoluminescence decay measurements for SAO:Eu,Dy (a) at 77K and for GYAGG:Ce,Cr (b) at 200K. Single exponential decay is observed with a 1.1μs lifetime for SAO:Eu,Dy and 52ns for GYAGG:Ce,Cr.
