# Supplementary material for: Unraveling Thermal Interactions in Lanthanide-Doped Phosphors: A Frequency-Domain Analysis Approach
Source: J Phys Chem Lett. 2026 Mar 4;17(11):3266–71. doi: 10.1021/acs.jpclett.5c04010 (PMC13007026; doi:10.1021/acs.jpclett.5c04010)
Supplement: Supplementary file 2 [file jz5c04010_si_002.pdf]

jz-2025-04010y.R1

Name: Peer Review Information for "Unraveling Thermal Interactions in Lanthanide-Doped Phosphors: A Frequency-Domain Analysis Approach"

First Round of Reviewer Comments

Reviewer: 1

Comments to the Author

The manuscript addresses the important problem of thermal quenching in lanthanide-doped phosphors, focusing on the respective roles of thermal crossover and thermal ionization. The work builds directly on the authors' previous report on frequency-domain luminescence analysis (Adv. Opt. Mater. 2025, 10.1002/adom.202501847) and extends this methodology to persistent-luminescence systems. In this context, the application of a frequency-domain, PersL-type analysis to delayed luminescence is methodologically relevant and provides valuable access to trap-related dynamics and carrier-release kinetics.

When combined with conventional steady-state and time-resolved photoluminescence and with band-structure considerations, the approach contributes to a more comprehensive experimental toolbox. However, the manuscript should be viewed as a methodological contribution rather than a definitive resolution of thermal crossover versus thermal ionization in lanthanide phosphors.

Major Comments

1. The introduction treats crossover and ionization as largely separable processes. In practice, these mechanisms often coexist and interact. The authors should clarify whether their frequency-domain approach is intended to fully disentangle these processes or rather to identify their relative contributions or dominant pathways over specific temperature ranges.

2. The manuscript would benefit from clearer contextualization of the frequency-domain approach relative to other dynamic or modulation-based luminescence techniques used to study thermal quenching and trapping phenomena, to properly position its methodological novelty.

3. The claim that standard PL quenching curves "fail to provide mechanistic insight" (page 3) is overly absolute. Stating that the "standard analysis" cannot distinguish the contributions of different quenching mechanisms requires clarification of what is meant by "standard." If "standard" refers strictly to steady-state I(T) measurements analysed using a single Arrhenius fit, then the statement is largely correct, as such an analysis is intrinsically underdetermined. In practice, however, experimental studies often go beyond this level of analysis. Temperature-dependent lifetimes, time-resolved decay components, and composition-dependent trends can already provide additional discriminating power, even when the results are presented in the form of quenching curves (see, for example, Chem. Mater. 2019, 31, 3851; 10.1021/acs.chemmater.8b05300). The authors should therefore clarify what they mean by "standard analysis" and adopt a more balanced formulation.

4. In the current formulation, Γ_{cross} is lumped together with Γ_{rad} and Γ_{nr} into a single Γ_{tot}. As a result, crossover is not separated from other prompt nonradiative channels; only ionization, which produces traps and delayed components, acquires a distinct dynamical signature. This limitation should be explicitly stated to avoid overinterpretation of the method's resolving power.

Minor Comments

Page 5: The statement that systems without thermal ionization exhibit "only one characteristic rate" is an approximation that should be explicitly acknowledged as such.

Page 6: The quantities Hr and Hi used in the Nyquist plot should be clearly defined.

Page 8: Additional details on the derivation and assumptions underlying Eq. 2 are needed.

Page 9: The fitted parameters obtained from Eqs. 4 and 5 should be compared with values reported in the literature to assess their physical plausibility.

Reviewer: 2

Comments to the Author

The authors present a very interesting and innovative approach to study the (delayed) excited state dynamics of two common persistent phosphors: SrAl2O4:Eu2+, Dy3+ and $(Gd_{0.33}Y_{0.67})_3Al_{2.4}Ga_{2.6}O_{12}$:Ce3+,Cr3+. It is shown that the conventional decay dynamics of the emitter works on a much different time scale than the trapping dynamics and that simultaneous tuning of temperature and frequency can help disentangle these two processes that both lead to a similar thermal quenching outcome for phosphors.

I generally think that this manuscript offers a fresh, new insight into T-dependent excited state dynamics by means of frequency-domain techniques. This has encountered a revival in the last few years and I think that this solid work will be appreciated by the community. Thus, I clearly see a match to the scope of J. Phys. Chem. Lett. However, a few points should be discussed/analyzed more carefully prior to final publication:

1) As far as I got, the whole analysis works so well with these two phosphors because the time scales of (essentially radiative) intrinsic decay and trapping is very different that makes it easy to distinguish the imaginary parts Hi in a frequency-domain spectrum. On top, trapping only becomes clearly relevant in these two materials at room temperature or even above.

What I struggle a bit with is the following: What if a phosphor is considered, for which a) a strong nonradiative quenching contribution accelerates intrinsic decay even at moderate temperatures or b) trapping may already occur at lower temperatures. In that case, the frequencies do not differ overly much anymore and the analysis becomes more complex. Does it yet offer a benefit for such delicate cases? That would be a real proof-of-concept example.

2) Based on point 1: It may be generally good to demonstrate the capability of this method for a challenging example or at least address what the authors would expect there (given the fact that these experiments are time-consuming indeed and the method generally works).

2) In principle, this analysis works based on very different time scales of the two processes of interest that give rise to different amplitudes in the decay dynamics. Given the fact that time-resolved methods have nowadays become extremely mature, it may be helpful for the readers to stress the benefits of the authors' frequency domain approach. In principle, one could also envision a T-dependent time-resolved experiment, in which related amplitudes of the two processes of interest (decay vs. trapping) can be resolved much better. Intensified CCD cameras offer such an option for example. They would allow measurements in literally no time, too.

Thus, I had some trouble finding explicit arguments why people should go for a frequency-based approach. Just to make my point clear: I do see the general feasibility of this methodology and think it offers a huge benefit, but the manuscript does not readily stress why it would be urgent to go for a frequency-based approach instead of a time-resolved one. There are methods to also assess the authors' problems in a time domain, as far as I could grasp. I am curious about the authors' opinion on this and eager to enter a scientific discussion here (also knowing that the authors have a high level of standard and can certainly assess this!).

Author's Response to Peer Review Comments:

INSTITUTO DE CIENCIA DE MATERIALES DE SEVILLA

Seville, February 17th 2026

Dear Editor,

Please find attached a revised version of the manuscript "Unraveling Thermal Interactions in Lanthanide-Doped Phosphors: A Frequency-Domain Analysis Approach" (Research Article, No jz-202504010y), by M. Romero et al. One reviewer fully supports publishing our manuscript, provided that we address a few details. We are glad that the reviewers fully support the publication of our

manuscript, provided that we address their comments, which we have done in order to improve the quality of our manuscript.

We have considered all the suggestions made by the reviewers and by the editorial office and have modified the manuscript accordingly. To make the changes easy to follow, they are highlighted in yellow in the new version and explained in the response letter. We believe that the overall quality of the manuscript has improved as a result of the review process, and we are grateful for this. We hope that the new version of our manuscript is now suitable for publication in your journal.

Sincerely,

Dr. Gabriel Lozano
Research Investigator of the Spanish National Research Council at the Institute of Materials Science of Seville

Response letter to reviewers

Response to Reviewer #1 (R1)

R1: The manuscript addresses the important problem of thermal quenching in lanthanide-doped phosphors, focusing on the respective roles of thermal crossover and thermal ionization. The work builds directly on the authors' previous report on frequency-domain luminescence analysis (Adv. Opt. Mater. 2025, 10.1002/adom.202501847) and extends this methodology to persistent-luminescence systems. In this context, the application of a frequency-domain, PersL-type analysis to delayed luminescence is methodologically relevant and provides valuable access to trap-related dynamics and carrier-release kinetics. When combined with conventional steady-state and time-resolved photoluminescence and with bandstructure considerations, the approach contributes to a more comprehensive experimental toolbox. However, the manuscript should be viewed as a methodological contribution rather than a definitive resolution of thermal crossover versus thermal ionization in lanthanide phosphors.
Authors (A): We would like to thank the reviewer for her/his positive appraisal. We are glad that she or he acknowledges the relevance of our approach and support the publication of our manuscript, provided that we address the following comments.

R1: 1. The introduction treats crossover and ionization as largely separable processes. In practice, these mechanisms often coexist and interact. The authors should clarify whether their frequency-domain

approach is intended to fully disentangle these processes or rather to identify their relative contributions or dominant pathways over specific temperature ranges.

A: We agree that thermal crossover and thermal ionization generally coexist and interact over broad temperature ranges. Our frequency-domain approach is not intended to isolate these mechanisms as completely independent processes but rather to identify and quantify their relative contributions within specific temperature regimes. Specifically, our method distinguishes thermal ionization and trapping from other decay channels, such as radiative decay and nonradiative thermal cross-relaxation. As we explained in the new version of the manuscript, our approach provides a quantitative assessment of the competition between these mechanisms, rather than strictly separating each contribution. We have further clarified this point in the new version of the manuscript.

R1: 2. The manuscript would benefit from clearer contextualization of the frequency-domain approach relative to other dynamic or modulation-based luminescence techniques used to study thermal quenching and trapping phenomena, to properly position its methodological novelty.

A: Modulation-based techniques were proposed some years ago in the context of determining the lifetime of light sources (e.g., Ann. Rev. Biophys. Bioeng. 1984. 13: 105-24, 10.1146/annurev.bb.13.060184.000541; Nat Protoc. 2018, 13, 1979–2004, 10.1038/s41596-018-0026-5) and rapidly became relevant for analyzing fluorescent lifetime imaging microscopy (e.g. Nature Methods 2023, 20, 965–967, 10.1038/s41592-02301906-y). Recently, these techniques have been employed for analyzing trapping mechanisms in persistent luminescent materials (Adv. Optical Mater. 2025, 13, e01847, 10.1002/adom.202501847) and metal halide perovskites (ACS Energy Lett. 2025, 10, 7, 3122–3131, 10.1021/acsenergylett.5c01102; EPJ Photovoltaics 2025, 16, 11, 10.1051/epjpv/2024045). However, to our knowledge, there have been no previous reports on using modulation-based luminescence techniques to study the thermal properties of emitters. As a matter of fact, the most common approach to study these physical interactions relies primarily on steady-state, temperature-dependent photoluminescence; time-resolved decay; thermoluminescence; or photoconductivity measurements (e.g., Phys. Rev. B 95, 014303, 10.1103/PhysRevB.95.014303; J. Phys. Chem. C 2015, 119, 25003−25008, 10.1021/acs.jpcc.5b08828; J. Appl. Phys. 2011, 110, 053102, 10.1063/1.3632069; J. Mater. Chem. C 2015, 3, 5642, 10.1039/c5tc00546a). Following the reviewer's advice, we have revised the introduction to provide better contextualization.

R1: 3. The claim that standard PL quenching curves "fail to provide mechanistic insight" (page 3) is overly absolute. Stating that the "standard analysis" cannot distinguish the contributions of different quenching mechanisms requires clarification of what is meant by "standard." If "standard" refers strictly to steady-state

I(T) measurements analysed using a single Arrhenius fit, then the statement is largely correct, as such an analysis is intrinsically underdetermined. In practice, however, experimental studies often go beyond this level of analysis. Temperature-dependent lifetimes, time-resolved decay components, and composition-dependent trends can already provide additional discriminating power, even when the results are presented in the form of quenching curves (see, for example, Chem. Mater. 2019, 31, 3851; 10.1021/acs.chemmater.8b05300). The authors should therefore clarify what they mean by "standard analysis" and adopt a more balanced formulation.

A: By "standard analysis," we refer specifically to steady-state photoluminescence intensity measurements as a function of temperature. These measurements are typically interpreted solely through simplified Arrhenius-type fits. This approach does not allow for the unambiguous discrimination of competing quenching mechanisms. We agree that a more complete experimental characterization provides additional mechanistic insight beyond simple quenching curves (e.g. Phys. Rev. B 95, 014303,

10.1103/PhysRevB.95.014303; J. Phys. Chem. C 2015, 119, 25003−25008, 10.1021/acs.jpcc.5b08828; J. Appl. Phys. 2011, 110, 053102,
10.1063/1.3632069; J. Mater. Chem. C, 2015, 3, 5642, 10.1039/c5tc00546a). In light of the reviewer's comment, we have revised the manuscript to clarify this point.

R1: 4. In the current formulation, Γ_{cross} is lumped together with Γ_{rad} and Γ_{nr} into a single Γ_{tot}. As a result, crossover is not separated from other prompt nonradiative channels; only ionization, which produces traps and delayed components, acquires a distinct dynamical signature. This limitation should be explicitly stated to avoid overinterpretation of the method's resolving power.

A: The referee is correct that, in our model, the thermal crossover rate, $\square_{cross}$, contributes to the total excitedstate emptying rate, $\square_{tot}$, along with the radiative decay rate, $\square_{rad}$, and other nonradiative channels, $\square_{nr}$. Consequently, crossover does not appear as an independent dynamic feature in the frequency response. However, thermal ionization introduces an additional pathway involving charge trapping and delayed recombination. This gives rise to a distinct low-frequency signature that can be directly resolved in the frequency domain. Therefore, our approach primarily separates ionization-related trapping dynamics from fast decay processes rather than fully isolating crossover from all other nonradiative channels. We have addressed this in the revised version of the manuscript, following the recommendation of the reviewer.

R1: Page 5: The statement that systems without thermal ionization exhibit "only one characteristic rate" is an approximation that should be explicitly acknowledged as such.

A: We agree with the reviewer that this statement is misleading and needs to be clarified. By "only one characteristic rate", we meant a single fingerprint in the frequency response. This behavior is observed in Figures 2b and 2e at low temperatures, where a single peak appears in the imaginary part of the transfer function (H_i) at a high frequency. This peak is associated with fast excited-state dynamics and corresponds to $\square_{tot}$, which is defined as the sum of radiative and non-radiative decay contributions, including thermal crossover. A second peak in the low-frequency range only appears when thermal ionization occurs, since the dynamics associated with charge detrapping are much slower. The temperatures at which this occurs are T>□240 K for SAO and T>□290 K for GYAGG. In this latter case, the system is governed by "two" rather than "one characteristic rate." We have clarified this point in the new version of the manuscript.

R1: Page 6: The quantities H_r and H_i used in the Nyquist plot should be clearly defined.

A: We thank the reviewer for pointing out this oversight. In the revised version, we have explicitly defined H_r and H_i, respectively, as the real and imaginary components of the transfer function of the luminescence response under modulated excitation.

R1: Page 8: Additional details on the derivation and assumptions underlying Eq. 2 are needed.

A: In the new version of the manuscript, we have included the derivation of Eq. 2 and provided more details about the underlying assumptions, following the reviewer's suggestion.

R1: Page 9: The fitted parameters obtained from Eqs. 4 and 5 should be compared with values reported in the literature to assess their physical plausibility.

A: We agree with the reviewer that a comparison with previously reported values is relevant. In fact, the obtained detrapping-related parameters (s_2 and E_2) fall within the typical ranges reported in the literature for similar persistent phosphors (e.g. J. Lumin. 2008, 128 (1), 173–184, 10.1016/j.jlumin.2007.07.006; J. Phys.

Chem. Lett. 2023, 14 (45), 10151–10157, 10.1021/acs.jpclett.3c02638.). However, reported values for the trapping barrier E_1 are scarce, and, to the best of our knowledge, this work provides the first experimental

estimate of the trapping frequency factor s_1. We have addressed this relevant point in the revised version of our manuscript.

Response to Reviewer #2 (R2)

R2: The authors present a very interesting and innovative approach to study the (delayed) excited state dynamics of two common persistent phosphors: $SrAl_2O_4:Eu^{2+}$, Dy^{3+} and $(Gd_{0.33}Y_{0.67})_3Al_{2.4}Ga_{2.6}O_{12}:Ce^{3+},Cr^{3+}$. It is shown that the conventional decay dynamics of the emitter works on a much different time scale than the trapping dynamics and that simultaneous tuning of temperature and frequency can help disentangle these two processes that both lead to a similar thermal quenching outcome for phosphors. I generally think that this manuscript offers a fresh, new insight into T-dependent excited state dynamics by means of frequency-domain techniques. This has encountered a revival in the last few years and I think that this solid work will be appreciated by the community. Thus, I clearly see a match to the scope of J. Phys. Chem. Lett. However, a few points should be discussed/analyzed more carefully prior to final publication.
A: We would like to thank the reviewer for her or his positive feedback. We are glad that he or she appreciates the potential of our approach and supports the publication of our manuscript, provided that we address the following comments.

R2: 1) As far as I got, the whole analysis works so well with these two phosphors because the time scales of (essentially radiative) intrinsic decay and trapping is very different that makes it easy to distinguish the imaginary parts H_i in a frequency-domain spectrum. On top, trapping only becomes clearly relevant in these two materials at room temperature or even above. What I struggle a bit with is the following: What if a phosphor is considered, for which a) a strong nonradiative quenching contribution accelerates intrinsic decay even at moderate temperatures or b) trapping may already occur at lower temperatures. In that case, the frequencies do not differ overly much anymore and the analysis becomes more complex. Does it yet offer a benefit for such delicate cases? That would be a real proof-of-concept example.
A: As the referee points out, frequency analysis allows to separate physical processes that occurs at different time scales. In the case of persistent luminescent materials, the emission is the result of the interplay between the excited-state dynamics (radiative and non-radiative decay and trapping) and slow charge detrapping. As she/he mentions, when the rates associated with the fast and slow processes become comparable, the frequency response may exhibit overlapping features. This would make the analysis more challenging, requiring data processing or advanced modeling to extract the system's characteristic rates. Regarding the extreme cases the reviewer highlighted, the method remains applicable. a) If nonradiative contributions dominate over trapping (i.e., p_1 << [illegible]$_{tot}$), the low-frequency peak associated with detrapping will appear weak in the frequency response and may be obscured by noise. In this case, the approach's potential is limited only by instrumental sensitivity. b) If detrapping occurs at lower temperatures or faster timescales, as it would happen in materials dominated by shallow traps, the analysis will be more difficult. This can be particularly relevant for systems with broad trap distributions, which may necessitate deconvolution to distinguish between fast and slow processes. We have commented on this in the new version of the manuscript.

R2: 2) Based on point 1: It may be generally good to demonstrate the capability of this method for a challenging example or at least address what the authors would expect there (given the fact that these experiments are time-consuming indeed and the method generally works).
A: In this study, we focused on widely studied state-of-the-art persistent phosphors with clearly distinct detrapping and excited-state decay dynamics. This allowed us to develop the methodology and demonstrate its quantitative power clearly. Note that standard detrapping rates are on the order of seconds, whereas decay rates range from milliseconds for Cr^{3+} to microseconds for Eu^{2+} to nanoseconds for Ce^{3+} in emitters that are typically used in persistent phosphors. Consequently, all of the persistent materials we have investigated thus far in the frequency domain exhibit clearly distinguishable high- and low-frequency peaks. Nevertheless, we look forward to testing our method with systems that have overlapping dynamics, which are inherently more challenging, as the reviewer suggested. This interesting point has been addressed in the new version of the manuscript.

R2: 3) In principle, this analysis works based on very different time scales of the two processes of interest that give rise to different amplitudes in the decay dynamics. Given the fact that time-resolved methods have nowadays become extremely mature, it may be helpful for the readers to stress the benefits of the authors' frequency domain approach. In principle, one could also envision a T-dependent time-resolved experiment, in which related amplitudes of the two processes of interest (decay vs. trapping) can be resolved much better. Intensified CCD cameras offer such an option for example. They would allow measurements in literally no time, too. Thus, I had some trouble finding explicit arguments why people should go for a frequency-based approach. Just to make my point clear: I do see the general feasibility of this methodology and think it offers a huge benefit, but the manuscript does not readily stress why it would be urgent to go for a frequency-based approach instead of a time-resolved one. There are methods to also assess the authors' problems in a time domain, as far as I could grasp. I am curious about the authors' opinion on this and eager to enter a scientific discussion here (also knowing that the authors have a high level of standard and can certainly assess this!). A: We appreciate the reviewer's comment and agree that a similar analysis could, in principle, be performed using time-domain approaches. However, time-domain measurements present two critical limitations compared to frequency-domain characterization: i) First, to the best of our knowledge, there is currently no system capable of capturing the fast dynamics associated with the excited state decay rate (ns–ms) and the much slower detrapping rate (seconds to hours) with comparable resolution in a single measurement. This challenge is naturally addressed in the frequency domain since it allows for accurate sampling of fast and slow processes in a single measurement. ii) Second, quantitative separation of the different processes in the time domain would require extensive data processing and modeling. Direct experimental access to the transfer function H in the frequency domain, on the other hand, enables straightforward determination of the system's intrinsic rates without requiring fitting methods. This allows us to obtain absolute quantities, such as trapping efficiency, which are inaccessible using time-domain methods. We have commented on this in the new version of our manuscript.
